# Reliability of reporting of HIV status and antiretroviral therapy usage during verbal autopsies: a large prospective study in rural Malawi

**DOI:** 10.3402/gha.v9.31084

**Published:** 2016-06-09

**Authors:** Estelle M. Mclean, Menard Chihana, Themba Mzembe, Olivier Koole, Lackson Kachiwanda, Judith R. Glynn, Basia Zaba, Moffat Nyirenda, Amelia C. Crampin

**Affiliations:** 1 Karonga Prevention Study, Chilumba, Karonga, Malawi; 2 Faculty of Epidemiology and Population Health, London School of Hygiene and Tropical Medicine, London, UK

**Keywords:** verbal autopsy, Africa, Malawi, cause of death, demographic surveillance, HIV, ART

## Abstract

**Objective:**

Verbal autopsies (VAs) are interviews with a relative or friend of the deceased; VAs are a technique used in surveillance sites in many countries with incomplete death certification. The goal of this study was to assess the accuracy and validity of data on HIV status and antiretroviral therapy (ART) usage reported in VAs and their influence on physician attribution of cause of death.

**Design:**

This was a prospective cohort study.

**Methods:**

The Karonga Health and Demographic Surveillance Site monitors demographic events in a population in a rural area of northern Malawi; a VA is attempted on all deaths reported. VAs are reviewed by clinicians, who, with additional HIV test information collected pre-mortem, assign a cause of death. We linked HIV/ART information reported by respondents during adult VAs to database information on HIV testing and ART use and analysed agreement using chi-square and kappa statistics. We used multivariable logistic regression to analyse factors associated with agreement.

**Results:**

From 2003 to 2014, out of a total of 1,952 VAs, 80% of respondents reported the HIV status of the deceased. In 2013–2014, this figure was 99%. Of those with an HIV status known to the study, there was 89% agreement on HIV status between the VA and pre-mortem data, higher for HIV-negative people (92%) than HIV-positive people (83%). There was 84% agreement on whether the deceased had started ART, and 72% of ART initiation dates matched within 1 year.

**Conclusions:**

In this population, HIV/ART information was often disclosed during a VA and matched well with other data sources. Reported HIV/ART status appears to be a reliable source of information to help classification of cause of death.

## Introduction

The WHO recommends collecting data on HIV/AIDS history in verbal autopsies (VAs) (1), but there have been few published studies of validation of HIV status reported by a proxy after the person has died. VAs are used to assign likely cause of death (2) for programmatic purposes in surveillance sites in many low-income settings where most deaths are not registered or attended by a medical professional. In some research settings, such as our own, the VA can provide an additional source of information on HIV and antiretroviral therapy (ART) in the community for use in analyses of demography and mortality. The HIV prevalence in Malawi was estimated to be 10.6% in 2010 (3), and several sites in the country have used VAs (4–6).

A VA is an interview with a relative or friend who nursed the deceased (or was present) during most of their final illness, with open-ended and structured questions about signs and symptoms, pre-existing conditions, and treatments. Likely causes of death are assigned by physicians (7) or computer models (e.g. InterVA or InSilicoVA) (8). As may be expected, VAs compare well to hospital diagnoses (9) but less well with physical autopsies (10). VAs have been validated to identify HIV/AIDS deaths using symptoms alone (11), but assigning cause of death in HIV-positive people is becoming more complex. Increasingly widespread ART usage and prolonged duration on treatment is changing the course of the illness and increasing the range of non-specific symptoms relating to side-effects and acceleration of degenerative conditions, which are also seen in HIV-negative people. In addition to the respondent's account of the deceased's illness, any information from the deceased's medical records, including HIV status and use of ART, is considered useful to physicians when assigning the cause of death; HIV diagnosis is also a variable used to assign cause of death in computer models (12). In many settings where VAs are used, obtaining official medical information/history may be difficult due to lack of resources to attend clinics and search through the records, as well as poor record-keeping (13). In our setting in Malawi, personal health records are held by the families, though in 2014 the deceased's health record was only available in 41% of VAs. Often the only information available is from the VA respondent.

In many communities, despite rapidly increasing availability of ART, HIV is still surrounded by stigma (14), making disclosure of a positive HIV status challenging (15). Predictors of disclosure do not follow clear trends (15). People are more likely to disclose a negative status than a positive one (16) but are more likely to disclose their positive status to their families when they are ill and indeed may not have any choice in the matter during a severe or terminal illness (17). Relatives or friends may also make assumptions about a person's HIV status, based on their appearance, clinic attendance, past perceived sexual behaviour, or knowledge of the HIV status of a living or deceased spouse (18). Onward disclosure outside the family, even after the person has died and during a confidential interview, may not be socially or culturally acceptable.

Given these issues, it is important to know whether HIV/ART information reported in a VA can be relied upon to assist in assigning likely cause of death, evaluating ART programme success, and contributing data to other community-level analyses.

In the Karonga Health and Demographic Surveillance Site (HDSS) in Malawi, data on HIV status (actual test records and self-report) and data on use of ART (clinic records and self-report) can be linked, through personal identifiers that have been assigned to consenting individuals, to VA data, which have been systematically collected since 2003. We compared data from these pre-mortem sources to estimate the reliability of VA respondent reports of HIV status and ART usage, as well as their influence on cause of death assignment.

## Methods

### Population and data

#### Population

The Karonga HDSS is well established (19). In brief, a population of about 39,000 people living in rural villages in northern Malawi has been under continuous surveillance since 2002. Births and deaths are captured monthly, and in- and out-migrations annually. The HDSS is run by the Karonga Prevention Study (KPS), which conducts many multi-round surveys (including on socio-economic status and HIV sero-surveys) and evaluates the impact of interventions (e.g. ART), using the HDSS as a sampling frame. The Karonga HDSS is a member of the ALPHA network for analysis of longitudinal, population-based HIV/AIDS data in Africa (alpha.lshtm.ac.uk) and the INDEPTH network (indepth-network.org).

#### VA data

Following the report of a death, a VA is carried out by a KPS medical assistant, usually between 2 and 6 weeks after the death. The questionnaire, which has closed and open-ended questions, has changed over time but is based on the WHO standard VA questionnaire (1). Since 2003, respondents have been asked about the presence of disease symptoms and medical history, including whether the deceased had ever been diagnosed with HIV/AIDS; in 2009 more detailed questions were added about dates of HIV testing and ART usage.

Each completed VA questionnaire is reviewed by two clinicians, who independently assign a likely cause of death; if they do not agree, a third reviewer (a physician) makes the decision. A three-level hierarchical system of coding is used. As it is frequently not possible to distinguish between AIDS and TB deaths, such deaths are coded as follows: Level 1 (broad group), *communicable disease*; Level 2 (sub-group), *AIDS/TB*; Level 3 (single specific categories), one of *unspecified*, *pulmonary TB*, *AIDS*, or *other specific unlisted*. The HIV status of the deceased may be available to the reviewer within the VA questionnaire (through specific questions or in the free-text narrative or summary of their medical record) or through access to the HIV test data (see below).

#### HIV test data

A household-based adult HIV sero-survey using rapid tests was carried out in selected clusters in the HDSS in 2005–2006, followed by four adult HIV sero-surveys across the HDSS population from 2007 to 2011. In the first round, 83% of the population consented to testing, but this figure decreased to 73% by the fourth round. Rapid tests were used, and the vast majority of people consented to be informed of the result. HIV testing with disclosure to participants has also been offered during other studies from 1988 onwards. HIV testing is otherwise available from several providers in the area (and testing has rapidly increased during the period covered by this analysis), and self-reported data on previous test history in any setting and HIV status (which could be reported as positive, negative, or unknown) are collected at the time of HIV testing and in other studies.

#### ART use data

ART was available at the main hospital (which is 70 km from the HDSS area) beginning in June 2005, at the rural hospital within the HDSS area from September 2006 onward, and at smaller clinics in the HDSS area starting in October 2010. KPS identified and tracked cohorts of consenting people initiated on ART in all clinics, capturing current and previous ART usage (20).

#### Socio-economic data

Data on each individual's schooling and employment status are collected during annual HDSS surveys. As indicators of socio-economic status, we used the highest education level and an employment score based on occupation and the reliability of the income, where *low* is the least skilled and reliable (e.g. piecework) and *high* is the most skilled and reliable (e.g. a government worker paid monthly). The majority of the population are self-employed subsistence farmers and were classified as *medium*.

### Statistical methods

We restricted the analysis to adults aged 15 years and over, as HIV test data were only available for this group. We compared known HIV/ART status and date of ART initiation (according to test/clinic records or self-report) with information reported at VA and looked at factors associated with agreement. We used chi-square tests and kappa statistics and developed logistic regression models using forwards stepwise techniques – starting with a basic model including the deceased's sex, age, and year of death and keeping additional variables in the model if a likelihood ratio test gave a *p*-value smaller than 0.1. Available variables included time between date of death and VA, place of death, socio-economic status of deceased, type of respondent, number of HIV tests known to KPS, number of AIDS signs and symptoms that have been shown to have moderate to high specificity for predicting HIV/AIDS deaths in VAs (11) (oral candidiasis, jaundice, herpes zoster, weight loss, wasting, ulcers/sores, diarrhoea for more than 1 month or cough for more than 3 weeks), and HIV and ART status captured by KPS pre-mortem. We also looked at the correlation between HIV status reported in the VA and physician coding of deaths as HIV/AIDS for those without HIV status available in the KPS database (no negative HIV test within 3 years and no positive tests). We used Stata 13 for all analyses.

### Ethics

Ethical approval was obtained from the National Health Sciences Research Committee of Malawi (protocols 419, 424, and 448), and the Ethics Committee of the London School of Hygiene and Tropical Medicine (protocols 5081, 5067, and 5214).

## Results

From 2003 to 2014, 2,023 adult (15 years and older) VAs were conducted. HIV/AIDS diagnosis was asked about in all but 71 of the VAs (3.5%). Additional questions on HIV test and ART start dates were added in 2009, so were only available for 772 (38.2%) VAs; 588 (76.2%) of these reported a latest HIV test date, and 151 (19.6%) an ART start date.

### VA respondent reporting knowing HIV status

Of 1,952 VA respondents asked, 79.9% (1,560) reported that they knew the deceased's HIV status; this proportion increased over time, from 144/300 (48.0%) in 2003/04 to 299/303 (98.7%) in 2013/14 (Table 1).

**Table 1:** Characteristics of deceased according to whether respondents reported HIV status^a^

		HIV status known		Univariate chi-square	Multivariable logistic regression^b^ (*n*=1,952)	
			
	Total	*n*	%	*p*	OR	*p*
Total	1,952	1,560	79.9			
Sex				0.012		
Male	947	779	82.3		1	
Female	1,005	781	77.7		0.7	0.011
Age group (years)				<0.001		
15–19	69	57	82.6		1	
20–29	213	144	67.6		0.5	0.066
30–44	503	358	71.2		0.6	0.23
45–59	342	269	78.7		0.9	0.766
60–74	369	309	83.7		1.3	0.471
75+	456	423	92.8		3.1	0.004
Year of death				<0.001	1.3	<0.001
2003–2004	300	144	48.0			
2005–2006	386	312	80.8			
2007–2008	344	240	69.8			
2009–2010	317	273	86.1			
2011–2012	302	292	96.7			
2013–2014	303	299	98.7			
Place of death				0.043	N/A	
Home	705	583	82.7			
Health facility	1,012	801	79.2			
Traditional healer	23	15	65.2			
Relative's house	89	65	73			
Other	121	94	77.7			
Missing^c^	2	2	100			
Highest schooling				<0.001	N/A	
None	259	217	83.8			
1–3 years of primary	394	344	87.3			
4–7 years of primary	466	373	80			
Primary completed	357	257	72			
JCE completed	168	133	79.2			
MSCE completed	207	148	71.5			
Missing^c^	101	88	87.1			
Rank of employment				<0.001	N/A	
Low	513	434	84.6			
Medium	1,170	929	79.4			
High	175	115	65.7			
Missing^c^	96	82	85.4			
Type of respondent				<0.001	N/A	
Parent	286	212	74.1			
Partner/sibling/child	1,138	936	82.2			
Other relative	485	386	79.6			
Non-relative	43	26	60.5			
AIDS symptoms				0.001	N/A	
One or fewer	1,293	1,061	82.1			
Two or more	650	493	75.8			
Missing^c^	9	6	66.7			
Latest KPS/self-reported HIV result				0.224	N/A	
Negative	563	529	94			
Positive	279	257	92.1			
Unknown	117	105	89.7			
Total number of HIV tests reported to KPS				<0.001	1.4	0.001
None	1,156	817	70.7			
One to two	479	438	91.4			
Three to four	255	244	95.7			
Five or more	62	61	98.4			
Time between death and interview				0.575	1.01	0.073
Within 6 weeks	1,412	1,119	79.2			
6 weeks to 6 months	439	356	81.1			
6–12 months	79	66	83.5			
Over 12 months	22	19	86.4			

KPS, Karonga Prevention Study; OR, Odds ratio; JCE, Junior Certificate of Education (taken after 2 years of secondary school); MSCE, Malawi School Certificate of Education (taken after 4 years of secondary school).

a Where data on HIV status were available from verbal autopsy, *n*=1,952.

b Multivariable logistic regression model included only variables with a result here; all variables included in model as continuous variables except where OR given for each level.

c Records with missing values were excluded from the analysis concerning that variable.

In a multivariable analysis, the VA respondent reporting that they knew the deceased's HIV status was associated with the sex of the deceased (adjusted odds ratio [aOR] for female *vs*. male=0.7, *p*=0.011), age of the deceased (aOR for 20–29 years *vs*. 15–19 years=0.5, *p*=0.066), year of death (aOR for each increase of 1 year=1.3, *p*<0.001), and number of HIV tests captured pre-mortem by KPS (aOR for each additional test=1.4, *p*<0.001) (Table 1).

### Agreement on HIV status

There were 959 VAs for which the VA respondent was asked about the deceased's HIV status and there was a linked HIV status (positive, negative, or unknown) captured by KPS pre-mortem; 993 had no linked pre-mortem data. According to the VA respondents, 251 of the 959 (26.2%) were HIV positive, while 279/959 (29.1%) were HIV positive according to pre-mortem data (Table 2).

**Table 2:** Comparing HIV status reported to KPS prior to death with HIV status reported by respondent in the VA^a^

		HIV status reported in VA			
	
HIV status reported by deceased to KPS		Negative	Positive	Unknown	Total
Negative	*n*	515	14	34	563
	(row%)	(91.5%)	(2.5%)	(6%)	(100%)
	(column%)				(58.7%)
Positive	*n*	25	232	22	279
	(row%)	(9%)	(83.2%)	(7.9%)	(100%)
	(column%)				(29.1%)
Unknown	*n*	100	5	12	117
	(row%)	(85.5%)	(4.3%)	(10.3%)	(100%)
	(column%)				(12.2%)
Total	*n*	640	251	68	959
	(row%)	(66.7%)	(26.2%)	(7.1%)	(100%)

KPS, Karonga Prevention Study; VA, verbal autopsy.

a Where data on HIV status were available from both VA and other KPS source, *n*=959.

Overall there was 79.1% agreement (kappa statistic=0.6, *p*<0.001). In a multivariable analysis of the 959, agreement was associated with the age of the deceased (aOR for increase=1.2, *p*=0.047), type of respondent (aOR for partner/sibling/child *vs*. parent=0.3, *p*=0.005 and aOR for other relative *vs*. parent=0.3, *p*=0.004), number of HIV tests reported to KPS (aOR for increase=1.3, *p*=0.005) and the nature of HIV status reported pre-mortem to KPS (aOR for positive *vs*. negative=0.6, *p*=0.03 and aOR for unknown *vs*. negative=0.02, *p*<0.001) (Table 3).

**Table 3:** Characteristics of deceased according to whether HIV status reported by respondent agrees with other information known to KPS^a^

		VA agree		Univariate chi-square	Multivariable logistic regression^b^ (*n*=959)	
			
	Total	*n*	%	*p*	OR	*p*
Total	959	759	79.1			
Sex				0.006		
Male	485	401	82.7		1	
Female	474	358	75.5		1	0.941
Age group (years)				<0.001	1.2	0.047
15–19	30	25	83.3			
20–29	100	82	82			
30–44	231	199	86.1			
45–59	171	140	81.9			
60–74	183	142	77.6			
75+	244	171	70.1			
Year of death				0.002	1	0.937
2003–2004	1	0	0			
2005–2006	36	31	86.1			
2007–2008	97	64	66			
2009–2010	268	208	77.6			
2011–2012	279	223	79.9			
2013–2014	278	233	83.8			
Place of death				0.009	N/A	
Home	302	220	72.8			
Health facility	542	450	83			
Traditional healer	12	8	66.7			
Relative's house	40	31	77.5			
Other	63	50	79.4			
Highest schooling				0.056	N/A	
None	127	86	67.7			
1–3 years of primary	183	140	76.5			
4–7 years of primary	221	174	78.7			
Primary completed	180	147	81.7			
JCE completed	89	72	80.9			
MSCE completed	94	77	81.9			
Missing^c^	65	63	96.9			
Rank of employment				0.041	N/A	
Low	227	165	72.7			
Medium	607	488	80.4			
High	61	45	73.8			
Missing^c^	64	61	95.3			
Type of respondent				<0.001		
Parent	129	115	89.1		1	
Partner/sibling/child	590	471	79.8		0.3	0.005
Other relative	223	158	70.9		0.3	0.004
Non-relative	17	15	88.2		2.7	0.333
AIDS symptoms				0.068	N/A	
One or fewer	663	514	77.5			
Two or more	295	244	82.7			
Missing^c^	1	1	100			
Total number of HIV tests reported to KPS				<0.001	1.3	0.005
None	163	58	35.6			
One to two	479	404	84.3			
Three to four	255	236	92.5			
Five or more	62	61	98.4			
HIV status reported pre-mortem to KPS				<0.001		
Negative	563	515	91.5		0	–
Positive	279	232	83.2		0.6	0.03
Unknown	117	12	10.3		0.02	<0.001
Time between death and interview				0.474		
Within 6 weeks	675	531	78.7			
6 weeks to 6 months	230	183	79.6			
6–12 months	45	39	86.7			
Over 12 months	9	6	66.7			

a Where data on HIV status available both from VA and other KPS source, *n*=959.

b Multivariable logistic regression model included only variables with a result here; all variables included in model as continuous variables except where OR given for each level.

c Records with missing values were excluded from the analysis concerning that variable.

Excluding those VAs where the informant reported to KPS that they didn't know the deceased's status left 842 people; agreement was then 88.7% (kappa=0.5, *p*<0.001). Of the 842, 563 were negative in KPS or self-reported tests, and 515 were also reported negative by the respondent (specificity of respondent report=91.4%). Two hundred seventy-nine were HIV positive according to KPS or self-reported HIV test, and 232 were also reported positive in the VA (sensitivity of respondent report=83.2%).

### Agreement on ART usage

There were 154 VAs on HIV-positive people with information on ART usage that could be linked to self-reports or clinic reports of ART initiation: overall there was 83.8% agreement (kappa statistic=0.4, *p*<0.001). In a multivariable analysis of the 154, agreement was associated with the presence of fewer AIDS symptoms (aOR for two or more *vs*. one or fewer=0.1, *p*=0.004), number of HIV tests reported to KPS (aOR for increase=1.6, *p*=0.069), and ART usage reported pre-mortem to KPS (OR for started *vs*. not=31.3, *p*<0.001) (Table 4).

**Table 4:** Characteristics of deceased according to whether ART usage reported by respondent agrees with other information known to KPS^a^

		VA agree		Univariate^b^ chi-square	Multivariable logistic regression^c^ (*n*=154)	
			
	Total	*n*	%	*p*	OR	*p*
Total	154	129	83.8			
Sex				0.212		
Male	81	65	80.2		1	
Female	73	64	87.7		2.3	0.154
Age group (years)				0.821	1.2	0.604
15–19	4	3	75			
20–29	17	14	82.4			
30–44	78	64	82.1			
45–59	46	39	84.8			
60–74	7	7	100			
75+	2	2	100			
Year of death				0.765	1.1	0.483
2007–2008	4	3	75			
2009–2010	56	47	83.9			
2011–2012	52	42	80.8			
2013–2014	42	37	88.1			
AIDS symptoms				0.101	0.1	0.004
One or fewer	66	59	89.4			
Two or more	88	70	79.5			
Total number of HIV tests reported to KPS				0.035	1.6	0.069
None	12	8	66.7			
One to two	82	65	79.3			
Three to four	43	39	90.7			
Five or more	17	17	100			
ART usage reported pre-mortem to KPS				<0.001	31.3	<0.001
Never started ART	28	13	46.4			
Started ART	126	116	92.1			

a Where data on ART usage available both from VA and other KPS source, *n*=154.

b Univariate analysis for the following variables not shown (all *p*>0.1): place of death, highest schooling, rank of employment, type of respondent, and time between death and VA.

c Multivariable logistic regression model included only variables with a result here; all variables included in model as continuous variables except where OR given for each level.

According to clinic reports or self-reports, 28 people had not started ART, and 13 were also reported as such in the VA (specificity of respondent report=46.4%). In all, 126 people had started ART; 116 were also reported as such in the VA (sensitivity of respondent report=92.1%). In addition, 31 VAs provided information on whether the deceased had started ART that could not be linked to any pre-mortem data source, and 10 were reported to have started ART.

### ART initiation dates reported

An ART initiation date was reported in 151 VAs, of which 115 (76.2%) could be linked to a clinic or self-reported start date: 83 (72.2%) of these matched within 1 year, 20 (17.4%) were more than 1 year after, and 12 (10.4%) were more than 1 year before.

### Influence of VA HIV status on cause of death assignment

**Fig. 1:**
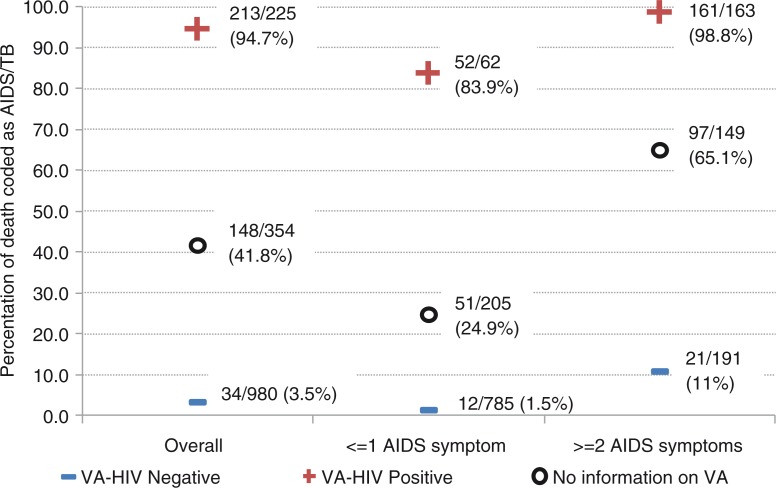
Percentage of deaths coded as HIV/AIDS where no HIV test data available to reviewers (*n*=1,559), showing influence of report from VA.

Of the 1,944 VAs with a likely cause of death assigned (79 [3.9%] did not have enough information to be able to assign a cause), 594 (30.6%) were coded as HIV/AIDS deaths. There were 1,559 VAs for which the reviewer had no KPS data on the deceased's HIV status (no negative HIV test within 3 years, and no positive tests), so cause of death would only be based on information from the VA questionnaire. In 354 cases there was no HIV status reported during the VA either, and 148 (41.8%) were coded as HIV/AIDS. In 225 cases, the respondent reported that the deceased was HIV positive and the death was coded as HIV/AIDS in 213 (94.7%), whereas in 980 cases a negative HIV status was reported and the death was coded as HIV/AIDS in only 34 (3.5%). This effect was also seen after stratifying the 1,559 according to whether they had AIDS symptoms: with one or fewer symptoms, a positive report from the VA increased the chance of a death to be coded as HIV/AIDS from 24.9 to 83.9%, and with two or more symptoms a negative report decreased this chance from 65.1 to 11.0% (Fig. 1).

## Discussion

Our study confirms the importance of HIV data reported by the VA respondent, showing how the respondent's report of the deceased's HIV status influences the way the physician reviewer assigns cause of death irrespective of symptoms: a death in a person with a given set of symptoms consistent with AIDS was much more likely to be coded as HIV/AIDS when the respondent said the deceased was HIV positive than when he or she was reported to be HIV negative or when the status was missing. HIV results (particularly positive) are also weighted heavily in computer models (12). VAs have often been shown to be fairly reliable compared to hospital notes (9), though a study in Ethiopia found that for HIV/AIDS and TB diagnoses the sensitivity was high but specificity lower and a number of false positives could be expected (21). This influence of VA HIV status on cause of death is likely to be found in other settings, which may have less complete or differential reporting of HIV/ART information in VAs, so it is important to know whether the HIV status reported by the VA respondent can be relied on.

In our study, the proportion of VA respondents reporting that they knew the deceased's HIV status was high and increased over time to 99%. The increase coincides with increased HIV testing (including increase of provider-initiated testing and counselling) and the rolling-out of ART in the area. Access to ART has been shown in other settings to normalise HIV and reduce stigma (22) so may make people more likely to discuss test results within families or to disclose HIV results to interviewers.

At 89%, overall agreement on HIV status between the VA respondent's report and pre-mortem KPS data sources was high, implying that, at least in this rural area of Malawi among those for whom we have a result, data on HIV status from VAs are reliable enough to be useful in interpreting VA. However, this must also be interpreted in the context of only a moderate kappa statistic of 0.5. Agreement was higher in HIV negatives than positives: disclosure (to or by the VA respondent) could be more likely when the result is negative, which has been found in other settings (15, 16, 23, 24), or the respondent could be less willing to report the deceased as HIV positive in a VA. A small number of people (14, 2.5%) were reported to have HIV/AIDS in the VA but only had records of negative HIV tests in the KPS data: these are likely to be true positives that had not yet been captured by KPS, as the most recent full sero-survey was in 2011.

Agreement on ART usage was also fairly high at 84% (though the kappa statistic was only moderate at 0.4), and 72% of ART initiation dates matched within 1 year. Some studies suggest that having started ART was associated with disclosure (25, 26), due to needing support during illness (27) and treatment but others have found the opposite effect (28). In Malawi it is recommended that people starting ART have a ‘treatment guardian’ (who attends the pre-ART counselling with the patient and is allowed to collect the medication on their behalf if required) (29), so it is to be expected that agreement would be high.

Agreement on HIV status was more likely if the deceased was male. Evidence on the association between gender and disclosure of HIV status is mixed, with many studies reporting no association (15, 25, 30, 31) and some reporting that women are less likely to disclose their status to anyone (24, 32). Unfortunately, the sex of the respondent was not recorded in our data.

In our data, increasing number of HIV tests reported by the deceased to KPS was associated with increased likelihood of the respondent reporting knowing the HIV status, as well as agreement on the status and on ART usage. Repeated voluntary HIV testing has been found to be associated with greater HIV knowledge, better health (33), and less risky sexual behaviour (34), which may also be linked to greater willingness to discuss HIV within the family.

We did not find any evidence that socio-economic status had any effect on reporting HIV/ART data in VAs; however the majority of the population are subsistence farmers, so there may not have been enough variation in socio-economic status to see an effect. The relationship between disclosure to a relative and socio-economic status is not consistent in the literature. One study in South Africa found that poorer women were less likely to disclose; however the opposite effect was seen in men (30). Some studies found no association between disclosure and education (25, 26), but others found that increased education was a predictor of disclosure (31, 35).

There was some evidence that if the respondent was the parent of the deceased, agreement on HIV status was more likely than if they were any other type of respondent. It has been reported that some people find it easier to disclose to female relatives (27) and that disclosing to the father is difficult (36) and less common than to the mother (26). A study in Ethiopia found that people were more likely to disclose to their partners but not to their children (35). We could not explore this as our respondent data are only available by the broad categories shown in Table 1: partner is combined with child and sibling and age and sex of the respondent were not collected.

The strength of our study of having a high number of VAs reporting HIV/ART information and being able to compare this information to other sources of pre-mortem data may make our results difficult to generalise to other settings, as regular sero-surveys and other studies create an artificial environment where more people know their status than would usually be the case and people are more used to taking part in interviews than in other areas. People who have taken part in more surveys will also have had more opportunity to report more HIV tests. About half of the VA records with HIV/ART information reported did not have any pre-mortem data to compare to, which made the numbers in some categories quite small, especially when looking at data on those who started ART, causing some difficulties in interpreting some of the results. People with no pre-mortem HIV/ART information may also have been different to those with information available, which could have introduced bias: deaths prior to 2007 (the first full sero-survey) would be less likely to have pre-mortem data, and pre-mortem data after 2011 (the final sero-survey) would come from smaller studies or ART clinics only, making them more likely to be HIV positive and seeking care. In addition, we allowed a person to be HIV negative for up to 3 years after a report of a negative test; however, they could have tested positive in that period prior to death so our pre-mortem data cannot be seen as infallible.

In conclusion, in this population, HIV/ART information was often reported during a VA in recent years and matched relatively well with other sources of data. Asking about HIV status and ART use is an important and, in this setting, reliable component of the VA. In other settings where reporting of HIV/ART information during a VA may be less common and more associated with background factors, bias might be introduced in assigning cause of death, which may affect the proportion of deaths attributed to HIV/AIDS in the community.

## Paper context

Verbal autopsies (VA) are commonly used to assign cause of death in low-resource settings for surveillance purposes. In areas affected by the AIDS pandemic, information on the deceased's HIV status and ART usage is vital in accurately assigning cause of death. Medical records are not always available so the VA-respondent's report maybe the only source of information. This paper assesses the reliability of VA-respondent reports of HIV/ART information in a rural African setting.
